# The Potential of Dendritic Cell Subsets in the Development of Personalized Immunotherapy for Cancer Treatment

**DOI:** 10.3390/cimb45100509

**Published:** 2023-10-01

**Authors:** Anna Valerevna Gorodilova, Kristina Viktorovna Kitaeva, Ivan Yurevich Filin, Yuri Pavlovich Mayasin, Chulpan Bulatovna Kharisova, Shaza S. Issa, Valeriya Vladimirovna Solovyeva, Albert Anatolyevich Rizvanov

**Affiliations:** 1 Institute of Fundamental Medicine and Biology, Kazan Federal University, 420008 Kazan, Russia; anagorodilova@yandex.ru (A.V.G.); krvkitaeva@kpfu.ru (K.V.K.); ivyfilin@kpfu.ru (I.Y.F.); mayasin_yuriy@mail.ru (Y.P.M.); harisovachulpan@gmail.com (C.B.K.); vavsoloveva@kpfu.ru (V.V.S.); 2 Department of Genetics and Biotechnology, St. Petersburg State University, 199034 St. Petersburg, Russia; st103070@student.spbu.ru

**Keywords:** dendritic cells, immunotherapy, clinical trials, tumor-associated antigen, cancer

## Abstract

Since the discovery of dendritic cells (DCs) in 1973 by Ralph Steinman, a tremendous amount of knowledge regarding these innate immunity cells has been accumulating. Their role in regulating both innate and adaptive immune processes is gradually being uncovered. DCs are proficient antigen-presenting cells capable of activating naive T-lymphocytes to initiate and generate effective anti-tumor responses. Although DC-based immunotherapy has not yielded significant results, the substantial number of ongoing clinical trials underscores the relevance of DC vaccines, particularly as adjunctive therapy or in combination with other treatment options. This review presents an overview of current knowledge regarding human DCs, their classification, and the functions of distinct DC populations. The stepwise process of developing therapeutic DC vaccines to treat oncological diseases is discussed, along with speculation on the potential of combined therapy approaches and the role of DC vaccines in modern immunotherapy.

## 1. Introduction

Dendritic cells (DCs) are proficient antigen-presenting cells (APCs) that play a pivotal role in initiating an adaptive immune response [1]. Among the diverse range of APCs in the human body, such as macrophages and B-lymphocytes, DCs are considered the most efficient in capturing antigens at the site of infection and delivering them to secondary lymphoid organs, where T-cell clustering takes place for subsequent antigen presentation and the activation of effector cells [2].

DCs are usually referred to as a link between innate and adaptive immunity. As part of the innate immune system, DCs contribute to the initiation of inflammatory processes while also playing a crucial role in activating the acquired immune response by presenting antigens on major histocompatibility complex (MHC) molecules [3]. Modern sequencing technologies have enabled the gradual characterization of the diversity of human DC subsets, with the determination of their exceptional role in shaping the immune response.

The main concept behind creating a vaccine based on DCs is to utilize their ability to activate and enhance the immune response against specific antigens [4]. Mature antigen-loaded DCs are capable of activating the immune system and directing it towards fighting tumors. Therefore, a DC-based vaccine allows us to increase the immune response against a specific antigen and improves the treatment effectiveness. One of the main advantages of this type of therapy is its low toxicity compared to other methods and its use as a safe adjuvant treatment method [5].

## 2. Biology of Dendritic Cells

Among other populations of immune cells, DCs are identified by their high expression of MHC II molecules and CD11c, which are considered necessary for their primary functions of antigen capture and subsequent processing in complex with MHC molecules [6]. To carry out the processes of the modulation of the immune response, DCs must migrate to the site of inflammation along the gradient of chemokine concentration. DCs express the chemokine receptors C-C type 5 and C-C type 6. The signaling axes CCR5-CCL5 and CCR6-CCL20, which include CCR5 and CCR6 and their ligands expressed by the tumor microenvironment (TME), are important for the successful recruitment of DCs to the TME [7]. DCs, which penetrate into the TME under cytokine gradient conditions, are capable of producing cytokines, which induce the migration and modulate the action of lymphocytes [8,9,10]. Additionally, DCs are able to effectively recruit NK cells into the TME [11] and activate them, particularly through the production of cytokines CXCL9 and CXCL10 [12].

DCs exist in two distinct physiological states in the human body. In tissues, DCs in a steady state or immature condition display low levels of costimulatory molecules and are incapable of activating naive T-lymphocytes [13]. Immature DCs also exhibit high endocytic capacity, high levels of adhesion molecules for tissue localization, and low levels of immune-stimulatory cytokines. Antigen capture processes performed by DCs are diverse and involve mechanisms such as phagocytosis [14], receptor-mediated endocytosis (lectin-dependent endocytosis, Toll-like receptor-mediated endocytosis) [15,16], and macropinocytosis [17].

The recognition of pathogen-associated molecular patterns (PAMPs) or damage-associated molecular patterns (DAMPs) serves as a stimulus for DCs to transition into a mature state. This transition is accompanied by changes in the expression of costimulatory molecules, integrin, and chemokine receptors, as well as the suppression of adhesion molecule expression [18]. All these processes contribute to the migration of DCs from the initial tissue site to the secondary lymphoid organs for the presentation of endogenous peptides via MHC I to CD8^+^ T-cells, and exogenous peptides in complex with MHC II to CD4^+^ T-lymphocytes [19]. In addition to antigen presentation, DCs can interact with T-cells through protein factors and costimulatory molecules such as CD80, CD86, OX40 ligand (OX40L), and CD70 [13,20].

## 3. Dendritic Cell Populations in the Human Body

**Figure 1:**
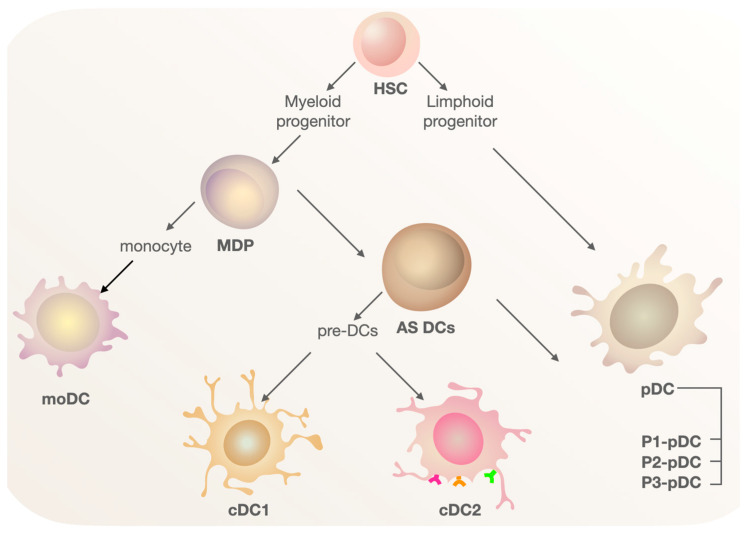
The scheme of the ontogenesis of DC populations. Myeloid and lymphoid precursors develop from hematopoietic stem cells. From the myeloid precursor, the macrophage DC progenitor (MDP) develops. The MDP further differentiates into monocytes and AXL^+^ SIGLEC6^+^ cells (AS DCs). AS DCs are capable of giving rise to both pre-DCs and pDC lineages. Pre-DCs are the precursors of cDC1 and cDC2. The pDC population, which originates from the lymphoid precursor, is also considered heterogeneous, with three distinct groups identified: P1-pDC, P2-pDC, P3-pDC.

DC populations in the human body demonstrate complex phenotypic and functional heterogeneity, which accounts for their broad functionality and substantial role in modulating immune responses. The following major subsets of DCs are observed in the human body: plasmacytoid DCs (pDCs), monocyte-derived DCs (moDCs), and conventional dendritic cells (cDCs) (Figure 1). These populations share a common myeloid precursor and are distinguished from other immune cells by their high expression of MHC II and CD11c molecules. However, during ontogenesis, they exhibit distinct repertoires of surface markers [6], which will be further discussed below. Interestingly, DC development is influenced by the microenvironment, especially in non-lymphohematopoietic tissue (lungs, skin, etc.), which additionally emphasizes their functional plasticity [21].

### 3.1. Conventional Dendritic Cells (cDCs)

cDCs are considered to play a crucial role in the activation of naive T-cells. Their main function is to capture and degrade protein antigens and present them as peptides in complex with MHC class I or II molecules [22]. cDCs are predominantly located in non-lymphoid tissues, especially in barrier tissues, which are the main sites of pathogen entry, where they perform antigen capture functions [23].

There are two main subsets of cDCs, cDC1 and cDC2, with each performing specific functions. The cDC1 subset is characterized by chemokine receptor XCR1 expression, with its ligand XCL1 secreted on the surfaces of CD8^+^ T-cells [24]. Among the distinctive set of specific markers, CLEC9A, involved in the uptake of necrotic cells; CD141, a cell adhesion molecule; and CADM1, CD103, CD8α, and BDCA-3, whose role is not fully understood, should be noted [25]. This subset is characterized by the expression of transcription factors BATF3 and IRF8 [26]. cDC1s are also major producers of IL-12, which is necessary for the differentiation of various T-cell populations [27]. cDC1s are also specialized in activating CD8^+^ naive T-cells through cross-presentation via MHC I molecules, playing a crucial role in anti-tumor and antiviral immune responses [24,28]. The presence of Toll-like receptor 3 (TLR3), an endosomal protein, is characteristic of cDC1s, and it is necessary for the recognition of double-stranded RNA, an intermediate in the replication of many viruses [29].

Subpopulation cDC2 is predominantly localized in secondary lymphoid tissue and is characterized by the expression of CD11b, CD1c, BDCA-1, and Dectin-1 surface markers [30,31]. Unlike cDC1, this subset neither expresses TLR3 nor is a major producer of IL-12. Physiologically, cDC2s induce the activation of naive CD4^+^ cells [30,32]. For example, in the induction of Th17 cells, cDC2s express IL-23 and IL-6 molecules, although the exact activation mechanisms are still being elucidated [18].

Using RNA sequencing technologies, it is possible to identify new subsets of DCs from those described above. The diversity of the precursors of the aforementioned populations at different stages of differentiation is increasingly taken into account. A. Villani’s group identified a population of DC precursors—AS DCs—characterized by the expression of AXL, SIGLEC1, and SIGLEC6 antigens, capable of giving rise to both cDC and pDC lineages [33]. P. See and others have identified a population of pre-DCs with an immunophenotypic profile of CD123^+^ CD33^+^ CD45RA^+^. Pre-DCs appear to be a later stage of differentiation from AS DCs and are precursors of cDC1 and cDC2, but not pDCs. Due to the presence of common markers, pre-DCs may be mistakenly classified as pDCs. Therefore, the secretion of IL-12 and activation of T-cells in pDCsare likely due to the presence of a small population of classical DC precursors. Pre-DCs differentiate towards CADM1^+^ CD1c^−^ pre-cDC1 and CADM1-CD1c^+^ pre-cDC2 [34].

### 3.2. Plasmacytoid Dendritic Cells (pDCs)

PDCs are characterized by the production of type I interferons (IFN-α), which is attributed to the endoplasmic reticulum and Golgi apparatus in their cellular structure [35]. Among the expressed receptors, CD11c, CD33, CD11b, and CD13 are absent, while GMDP, CD123 (IL-3R), and CD45RA are observed. Receptors involved in IFN-α production include CD303 (CLEC4C; BDCA-2), CD304 (neuropilin; BDCA-4), CD85k (ILT3), CD85g (ILT7), Fc ε R1, BTLA, CD358, and CD300A [36,37,38]. pDCs are primarily involved in the detection of viral infections and do not play a significant role in stimulating naive T-cells. pDCs express TLR7 and TLR9 in order to recognize nucleic acid molecules, which are key receptors in recognizing endosomal patterns [39,40,41].

The production of IFN-α is not the only function of this subset. Alculumbre et al. were able to characterize three subsets of pDCs from the general population based on the expression of the costimulatory and inhibitory molecules PD-L1 and CD80. After removing AS DCs that could have influenced the experimental results, the multifunctional property of pDCs was determined. P1-pDCs (PD-L1^+^CD80^−^) displayed a pronounced plasmacytoid morphology, and they are the main producers of IFN-α. P3-pDCs (PD-L1^−^CD80^+^) acquire a dendritic morphology and adaptive immune functions. P2-pDCs (PD-L1^+^CD80^+^) promote T-cell activation and differentiation towards Th2 cells.

### 3.3. Monocyte-Derived Dendritic Cells (moDCs)

MoDCs are derived from monocytes, and they undergo functional changes in inflammatory foci. Similar to other DC populations, moDCs can also transport antigens to lymphoid tissue [42]. However, the process of differentiation can be significantly influenced by the TME conditions, such as increased lactate levels, a byproduct of tumor cell metabolism, which has a negative effect on the increase in moDCs in the TME [43]. In addition, high levels of lactate also negatively affect the production of anti-inflammatory cytokines and the cytotoxic properties of T-cells and NK cells [44,45]. MoDCs can be easily obtained from human peripheral blood monocytes by laboratory generation [46]. Experiments show that moDCs are weaker stimulators of T-cell activation; nevertheless, upon migration to the lymph nodes, moDCs can transfer captured antigens from the periphery and present them to resident DCs in the lymphoid organs. This cell population exhibits heterogeneous expression of markers including CD13, CD33, CD11b, CD11c, CD172a, S100A8/9, CCR2, CD1c, CD1a, Fc εR1, IRF4, and ZBTB46. Similar to classical DCs, they express CD11c and MHC II [47]. moDCs are effective and perform their functions efficiently in vivo, by interacting with cDCs and responding to the microenvironment’s cytokine profile. However, in vitro studies of moDC-based vaccines have often yielded disappointing results, which can vary depending on the utilized activation and differentiation methods [48].

## 4. In Vitro DC Vaccine Design

### 4.1. DC Differentiation

**Figure 2:**
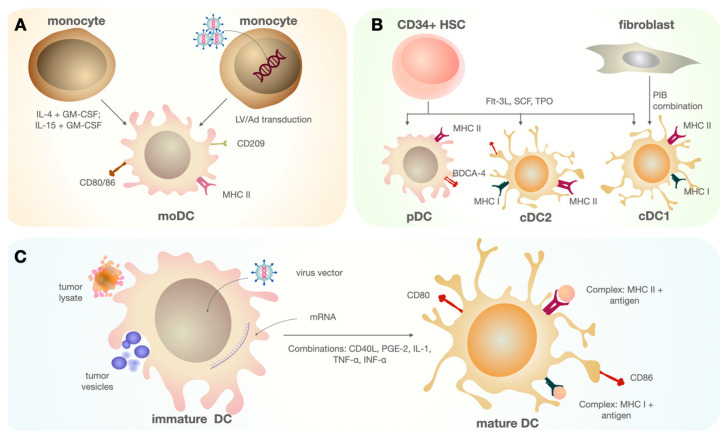
DCs can be created by various methods in vitro. (**A**) MoDCs are obtained through directed differentiation from CD14^+^ monocytes using various cytokine combinations, such as IL-4, GM-CSF, IFN-α, IL-15, etc. Resulting cells express several surface markers that are characteristic of moDCs and necessary for antigen presentation [53]. (**B**) A combination of three main subsets of DCs—cDC1, cDC2, and pDC—can be obtained from HSC CD34^+^ using FMS-like tyrosine kinase 3 ligand (FLT3L), thrombopoietin (TPO), and stem cell factor (SCF). A population similar in marker composition to cDC1 can be obtained through the direct reprogramming of fibroblasts transduced with the transcription factor set PU.1 + IRF8 + BATF3 (PIB) [54]. (**C**) The process of antigen internalization and DC activation occurs, where the antigens can be tumor vesicles, inactivated tumor cells, or the lysates of tumor cells. Antigen capture occurs, mainly through receptor-mediated phagocytosis mechanisms (lectin-dependent endocytosis, Toll-like receptor endocytosis, and macropinocytosis). One method involves transduction, where the DNA sequence encodes for antigens. An electroporation procedure is used to internalize mRNA molecules. In addition to antigen processing in complex with MHC molecules, a combination of activating molecules serves as a stimulus for maturation under in vitro conditions. The activated (mature) state of DCs is characterized by changes in the expression of costimulatory molecules (CD80, CD86) and integrin and chemokine receptors (CCR7), as well as the suppression of adhesion molecule expression.

DC vaccine development is a multi-step process, with its efficacy influenced by factors such as the culture conditions, antigen selection, and additional parameters. The first step in DC vaccine development is obtaining a sufficient number of cells for further manipulations. Current approaches to generating DCs mainly focus on in vitro differentiation from CD14^+^ or CD34^+^ monocyte precursors [49,50,51,52] (Figure 2). This direction is driven by the fact that DCs, circulating in the body, are a small population of immune cells, and isolating them in the required quantity to achieve therapeutic effects is rather challenging.

It is worth noting that ex-vivo-generated cells exhibit transcriptional profile differences compared to their in vivo counterparts [55,56]. This could potentially be the cause of the limited therapeutic efficacy observed in DC vaccines utilizing these strategies. Nevertheless, many preclinical and clinical trials have demonstrated the ability of generated DCs to activate T-cells and secrete anti-inflammatory cytokines, such as IL-12 [57].

#### 4.1.1. DCs Derived from Monocytes (moDCs)

**Figure 3:**
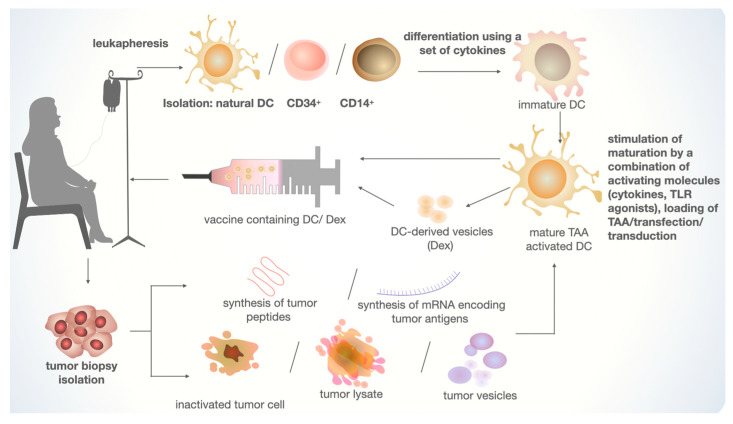
The main stages of creating a personalized DC-based vaccine for cancer treatment. Natural DCs, CD14^+^ monocytes, or CD34^+^ are isolated from leukapheresis material. CD14^+^ and CD34^+^ cells are differentiated into immature DCs. For TAA, tumor material is isolated, which can be used to construct the necessary antigen. Mature DCs are obtained using a combination of activating molecules and loading TAA. The ready-made injection consists of mature TAA-activated DCs or vesicles obtained from activated DCs (Dex).

MoDCs are derived through the directed differentiation of monocytes isolated from the peripheral blood mononuclear cell fraction (PBMC), obtained from whole blood or leukapheresis [58,59] (Figure 3). Isolation is commonly performed using plastic adherence, positive selection using antibody-coated magnetic beads, or flow cytometry. This method represents the most commonly used approach among published articles [60,61,62,63,64]. CD14^+^ monocytes are differentiated into immature DCs over several days, alongside various factors, with the combination of IL-4 and granulocyte–macrophage colony-stimulating factor (GM-CSF) being the “gold standard” [65,66]. However, other combinations of different cytokines have also been tested. DCs differentiated in the presence of GM-CSF and IL-15 have shown to be the most effective inducers of Th17 responses [67]. The combination of GM-CSF and IFN-α also contributes to the activation of effector CD8^+^ lymphocytes and Th1 cells [68]. Subsequently, immature moDCs are loaded with antigen and matured using a set of factors. After 1–2 days, mature moDCs present as cells loaded with tumor-associated antigen (TAA), which are then cryopreserved and thawed as needed [59]. Although this method is considered to be time-consuming, it is, however, being currently used in several medical institutions.

The use of autologous DCs is considered a priority approach, as it avoids the immune rejection of the cells. Despite the explicit advantages of autologous therapy, allogeneic DC therapy also has been tested in several clinical trials [69]. Allogeneic DCs represent an attractive material, as the donor’s immune system is not compromised due to oncological conditions. However, this approach can be challenging given that it requires careful donor selection.

#### 4.1.2. DCs Derived from CD34^+^ Progenitors

Another approach to generating DCs involves differentiating CD34^+^ hematopoietic stem progenitor cells (HSPCs). By combining specific factors, certain populations of DCs can be obtained. For example, in several studies, the generation of cDC1s, the most efficient in antigen cross-presentation, has been demonstrated using a combination of recombinant FLT3L, SCF, GM-CSF, and IL-4 [70,71]. Generated cells exhibited the phenotype of true cDC1s: CD141^+^ CLEC9A^+^ XCR1^+^. However, obtaining an adequate number of cells to achieve therapeutic effects and multiple infusions remains a challenge [72]. Some studies have reported an up to 20-fold increase in the yield of generated cDC1s when co-cultured with HSPCs and the OP9 cell line, compared to classical methods [73,74].

#### 4.1.3. Genetic Reprogramming in DCs

Another approach to obtaining DCs involves genetic reprogramming, which, in theory, can allow the generation of the desired DC population, depending on the designed genetic cassette. Using a lentiviral vector encoding GM-CSF, IL-4, and melanoma-associated antigen (TRP2), it has been possible to differentiate CD14^+^ monocytes into moDCs loaded with the tumor antigen TRP2 from melanoma [75]. A similar approach has been used to generate induced cDC1s from fibroblasts by transducing a lentiviral vector that induces the expression of key transcription factors for cDC1s: PU.1, IRF8, and BATF3 [76,77].

### 4.2. DC Maturation

The next rational step in creating a DC vaccine is the process of DC maturation. This process involves antigen loading and the activation of DCs using factors that influence their physiological processes. There is currently no consensus on the optimal composition of activating molecules. These can include cytokines such as TNF-α, IFN-γ, Toll-like receptor (TLR) agonists (e.g., LPS), and agonistic recombinant proteins (e.g., CD40L). It should be noted that IFN-α is included in the cytokine combination in order to mimic viral infection [78]. Several approaches have been developed for the ex vivo generation of therapeutic DC vaccines, involving the acquisition of TAA. Such approaches include synthesized tumor peptides [79], full-length proteins, tumor heat shock proteins [80], autologous tumor cells (lysates and inactivated cells) [81,82], the introduction of mRNA encoding tumor antigens [83], and tumor vesicles [84]. All antigen-loading methods have shown efficacy under in vitro conditions, considering the combination with maturation factors. The use of tumor lysates is considered the most common approach to loading tumor cells [85,86]. A significant advantage of this method is enabling the loading of DCs with a polyantigen complex, including neo-antigens, specific to the patient’s tumor, due to the cellular heterogeneity of the malignancy.

An alternative approach to loading DCs would be the in vivo delivery of antigens to DCs, using liposomes [87], genetic vectors [88], or the fusion of antigens with monoclonal antibodies [89]. In this case, liposomes serve as a delivery method, for example, of RNA molecules encoding tumor antigens. This method leads to the activation of DCs in situ through endocytosis mechanisms [87]. Adeno-associated viruses carrying an antigen sequence can also act as viral delivery agents [88]. Liposomes conjugated with antibodies on their surfaces can also serve as an alternative antigen delivery method, which can enhance specific binding to target cells [89]. A proper combination of antigens and activating molecules, which do not induce immunosuppression or immune tolerance, would ensure optimal DC maturation and the subsequent priming of T-cells.

### 4.3. Vesicles Derived from Dendritic Cells

It is known that extracellular vesicles (EVs) released by immune cells modulate cell interactions in the TME [90]. For example, EVs can inhibit tumor growth, stimulate an immune response against malignant cells, and improve the infiltration of other immune cells into the TME [91]. Additionally, EVs can transmit information between immune cells, allowing them to coordinate their actions and enhance the immune response against the tumor [92]. Therefore, one of the new approaches to oncotherapy is the use of vesicles obtained from dendritic cells (Dex), which possess the properties of stem cells and have several advantages. The utilization of Dex as an alternative antigen delivery method, and their involvement in the in vivo activation of effector cells, is currently considered a promising direction in immunotherapy [93].

The process of Dex production involves all previous stages of DC creation, with the final step being the isolation of these structures from the supernatant through sequential centrifugation. Dex is naturally secreted by DCs and, similar to the parent cell, possesses a bilayer lipid membrane with a characteristic repertoire of protein molecules, including MHC I and MHC II, as well as costimulatory molecules such as CD80 and CD86, necessary for the interaction and activation of CD8^+^ and CD4^+^ T-cells [94]. Additionally, the Dex membrane expresses intercellular adhesion molecule 1 (ICAM-1) [45]. Research also indicates that Dex contains various cytoplasmic proteins and microRNAs [95]. As extracellular structures, Dex are less susceptible to tumor immunosuppressive mechanisms, suggesting a potentially more effective T-cell response [96]. Moreover, due to their stable configuration resembling exosomes and other extracellular vesicles, Dex can be stored frozen for at least six months [97].

### 4.4. Adaptive Transfer of DC Vaccines

In the context of in vivo conditions, DCs, following successful activation, migrate to the lymph nodes, via chemokine gradient, in order to interact with T-cells. Therefore, choosing a delivery method for autologous or allogeneic DC injections is crucial in achieving the required therapeutic effects. Intradermal injections of labeled DCs have shown that only approximately 2–4% of DCs migrate to the draining lymph nodes. Remaining cells perish at the injection site and are subsequently eliminated by immune cells (macrophages). However, W. Joost Lesterhuis et al. conducted a study comparing different methods of DC administration. In the study, it was demonstrated that the subcutaneous injection of DCs leads to the higher induction of anti-tumor properties in T-cells [98]. These findings can be explained by the fact that only the most mature and differentiated cells reach the lymph nodes. In intranodal injection, DCs are delivered directly to the site of interaction with lymphocytes. Nonetheless, this method does not achieve an optimal response, compared to subcutaneous injection, which may be attributed to the administration of activated and non-activated DCs, as well as non-viable vaccine cells [99]. Direct injection into the lymph nodes eliminates the loss of non-migratory cells; however, this approach requires precise manipulation control.

## 5. Application of DC Vaccines in Cancer Therapy

Currently, a wealth of preclinical research results has accumulated and been published, demonstrating the anti-tumor potential of DC vaccines. Despite progression in DC development from different precursors, and the utilization of TAA and activation factor combinations, as mentioned earlier, DC vaccines have shown discouraging results in clinical practice. Undoubtedly, one of DC vaccines’ advantages is the rare occurrence of third–fourth-grade adverse effects, as demonstrated in numerous clinical trials. Most side effects are minimal and characterized by first–second-grade symptoms, such as weakness, irritation at the injection site, and flu-like symptoms [100]. Toxic effects of the third–fourth grade have been reported in some published clinical trials and are likely to be associated with the therapeutic combination used [64].

### 5.1. Preclinical Studies

The diversity of preclinical studies aimed at assessing the effectiveness of DC-based vaccines demonstrates the current and future directions of development in this field. We consider several types of studies that reflect general trends in DC vaccine development.

Accumulating data suggest that DC vaccines, based on cDC1s, are more effective in priming T-cell responses, compared to similar moDC therapies. In their study, Stephen Ferris et al. evaluated the induction of T-cell responses by moDCs of bone marrow origin and generated cDC1s using mouse models. The study compared the ability of moDCs and cDC1s to directly prime T-cells in lymphatic vessels, without natural DCs’ involvement. *Irf8*^+^*32*^−/−^ mice, which lack endogenous cDC1s, were used for this purpose. Although the cross-presentation of antigens was demonstrated in in vitro models, the authors concluded that moDC injection in *Irf8*^+^*32*^−/−^ mouse xenotransplant models did not lead to tumor-specific responses without the involvement of cDCs [101]. The results of preclinical studies, involving various combinations of DC-based therapies, are presented in Table 1.

Shin-Wha Lee et al. found that a therapy approach using CD8α^+^ DCs, induced from HSCs, similar to the population of human CD141^+^ DCs, not only promotes tumor regression but also contributes to a higher level of immune-stimulating cells such as CD4^+^, CD8^+^, and CD11c^+^, as well as a lower level of the immunosuppressive cytokine IL-10 at lower therapeutic doses, compared to moDC therapy in a mouse model [102]. This, therefore, emphasizes the relevance of developing therapeutic vaccines based on cDCs. The current preclinical development of cDC-based vaccines aims not only to demonstrate differences, but also to optimize the protocol of obtaining cDCs from HSCs with maximal yields and a complete immunophenotypic marker set. Yuanzhi Bian et al. established the synergistic role of IFN-γ and TLR agonists in activating an immortalized mouse DC cell line (JAWSII (ATCC^®^ CRL-11904™, Manassas, VA, USA). Their research established a significant difference in the percentage of activated DCs treated with IFN-γ+poly I:C (polyinosinic-polycytidylic acid, a TLR agonist), compared to DCs treated with IFN-γ alone, indicating IFN-γ involvement in TLR signaling pathways upon their co-administration [103]. Mariana Oliveira et al. found that inhibiting signal transmission through WASp and Arp2/3, using a small molecule called CK666, promotes cross-presentation by reducing phagosomal acidification, resulting in antigen release into the cytoplasm. As a result, TAA presentation is mediated by MHC I molecules instead of MHC II molecules, leading to a higher level of proliferation of specific CD8^+^ T-cells in vitro and in vivo and the prolonged survival of mice receiving CK666-treated DCs [104].

**Table 1:** Results of some preclinical studies based on DC therapy.

Therapy	Model for Research	Results	References
Comparison of moDC-based therapy and cDC1-based therapy	*Irf8*^+^*32*^−/−^, *Batf3*^−/−^ mice C57BL/6, CD45.2^+^ *Irf8*^+^*32*^−/−^, mice with subcutaneous methylcholanthrene (MCA)-induced fibrosarcoma injections	Lack of tumor-specific response in the therapy of moDCs in *Irf8^+^32*^−/−^ mice	[101]
Comparison of CD8α^+^DC-based therapy and moDC-based therapy	C57BL/6 mice with orthotopic model of ID8 cancer	Reduced volume of ascites in both groups Decreased level of regulatory T-cells (Treg), IL-10, increased expression of CD3, CD4, CD8, and CD11c markers in the CD8α^+^ DC group	[102]
DC-based therapy + inhibitor Arp2/3 CK666	CD45.2 WT, OT-I and CD45.1 (Ly5.1) mice	The combination of DCs and CK666 inhibitor led to a reduction in phagosomal acidification and an increase in CD8^+^ T-cell proliferation, compared to the control group	[104]
bmDC therapy + DNA vaccine	Human MUC1 transgenic mice	Tumor regression was observed only in mice receiving therapy with bmDCs + DNA vaccine	[105]
DC-based vaccine + αPD-1	C3H/HeJ mice by transplanting murine MBT-2 bladder cancer cells	In the group “DC + αPD-1”, there was higher survival, IFN-γ production, and frequency of CD8^+^ and CD4^+^ T-cells in the spleen	[106]

Retno Murwanti et al. presented data on combined therapy using a DNA vaccine along with DCs derived from bone marrow (bmDCs). The DNA vaccine targeting MUC1, a tumor-associated antigen, and autologous bmDCs was tested as a monotherapy in human MUC1 transgenic mice with colorectal tumors. However, results showed that tumor regression was only achieved through the combination of the DNA vaccine and bmDCs. Accordingly, the authors highlighted bmDCs’ crucial contribution in enhancing the anti-tumor immune response in combination with a DNA vaccine designed for a specific target molecule [105].

Soyeon Lim et al. found that the combination of a DC vaccine loaded with lysate and an anti-PD-1 antibody (αPD-1) had a more pronounced therapeutic effect, compared to mice receiving either DC therapy alone or αPD-1 in a mouse model of bladder cancer. In the study, increased secretion of IFN-γ and splenocyte cytotoxicity were also found in mice [106]. Felipe Cezar de Mato tested the influence of peptides, isolated from spider venom, on the modulation of mouse DCs in vitro, based on previous data on the cytotoxic properties of these peptides on glioblastoma cells. The study results showed statistically significant differences in the expression of the costimulatory molecule CD86, when using the peptide and tumor cell lysate, compared to a DC + lysate, as well as the increased secretion of some proinflammatory cytokines [107].

### 5.2. Clinical Studies

To date, a multitude of clinical trials have used different DC vaccines, with the majority of them utilizing monocyte-derived DCs for loading with antigens [60,62,108,109], while only a few highlight the use of neoantigens [61]. Some studies employ DCs of natural myeloid origin [110]; however, the method of generating DCs from monocytes is considered the most commonly encountered. Selective clinical trials will be discussed further, as they reflect general trends in vaccine development and demonstrate patient outcomes.

#### 5.2.1. DC Progenitor-Based Therapy

At the end of 2022, data from phase 3 clinical trials of therapy, under the registered trademark DCVax-L—autologous mature DCs loaded with tumor lysate for the treatment of glioma (NCT00045968)—were published. PBMCs were obtained by leukapheresis and then cultured in the presence of GM-CSF and IL-4 cytokines. Antigen loading was performed using a tumor lysate obtained from tumor resection [111]. The phase 3 trials involved 331 patients, of whom 232 received DCVax-L injections, along with standard temozolomide treatment, and 99 patients were in the placebo group. The study aimed to analyze the vaccine effectiveness and its impact on the survival of patients with newly diagnosed glioblastoma (nGBM) or its recurrent form (rGBM). The study, which started in 2007, showed a statistically significant increase in patient survival for both NDGB and RGB patients. The median overall survival (mOS) for 232 NDGB patients receiving DCVax-L was 19.3 (95% confidence interval (CI) 17.5–21.3) months, compared to 16.5 (95% CI 16.0–17.5) months in the control group (98% CI 0.00–0.94). Among 64 RGB patients receiving DCVax-L, the mOS was 13.2 (95% CI 9.7–16.8) months after recurrence, compared to 7.8 (95% CI 7.2–8.2) months in the control group (98% CI) [60]. Clinical trial results based on personalized DC vaccines are presented in Table 2.

Nicholas J Vogelzang and colleagues demonstrated the results of phase 3 of a completed VIABLE study. The study tested the efficacy of autologous DCs, combined with docetaxel and prednisone, compared to a placebo group receiving only standard chemotherapy (NCT02111577), for 1182 men with metastatic castration-resistant prostate cancer. The vaccine contained moDCs loaded with inactivated human prostate adenocarcinoma cell line cells (LNCaP). Despite promising results from phase 1/2 clinical trials, prior to VIABLE, the mOS was 23.9% in the treatment group and 24.3% in the placebo group, indicating no difference in overall survival between the two groups [112]. The authors also highlighted a lack of adverse events (AE) in patients. Such results were attributed to the terminal stage of cancer in many patients, and the fact that the mOS rate in patients who had been receiving abiraterone or enzalutamide prior to the trial was significantly lower, indicating possible drug resistance [63].

Trials involving DC transduction for antigen processing are of particular interest. A phase 1 clinical trial (NCT01730118) tested the therapeutic efficacy of autologous DCs, transduced with an adenovirus expressing human epidermal growth factor receptor 2 (HEP2), for patients with metastatic solid tumors overexpressing HEP2. These patients had either progressive disease after standard treatment or no evidence of disease after tumor resection. Thirty-three patients were included in the study and divided into several groups, with each receiving different amounts of DCs. One patient achieved a complete response (CR), one achieved a partial response (PR), and five patients achieved disease stabilization (DS). Significantly, the study showed no AE with transduced DC therapy [109]. In the study NCT01826877, DCs delivering antigens via an adenoviral vector were also tested. No significant results were obtained in the study, with only one patient completing the treatment and achieving DS after 27 months [108]. Lisa H. Butterfield and colleagues investigated the effect of transduced DNA vaccines on patients with melanoma, in combination with intravenously administered IFN-α. The vaccine was constructed against three commonly known melanoma antigens (tyrosinase, MART-1, and MAGE-A6), in order to stimulate polyclonal CD8^+^ and CD4^+^ responses. Two CR, eight cases of stable disease (SD), and 14 cases of disease progression (PD) were recorded. In 51% of patients (18 out of 35), first–fourth-grade AE were observed; however, second–fourth-grade AE were likely associated with the use of IFN-α. Most participants showed an enhancement in tumor-specific CD8^+^ and CD4^+^ T-cell responses, which did not correlate with increased levels of IL-12. Moreover, it is worth noting that adding IFN-α did not improve the immune or clinical response in this trial [113].

As an example of a clinical trial based on a neoantigen DNA vaccine, the study by Zhenyu Ding et al. (NCT02956551) can be considered. For 12 patients with non-small cell lung cancer, DCs loaded with neoantigen peptides were used as immunotherapy in combination with cyclophosphamide treatment. In order to obtain neoantigens, the study authors performed RNA sequencing, along with the whole exome sequencing of DNA from patient biopsy material. The method of obtaining monocyte-derived DCs was chosen quite classically (leukapheresis + IL-4 and GM-CSF). Along with DC vaccination, patients received maintenance therapy, such as ipilimumab, radiation therapy, or chemotherapy, as the disease progressed. In the latest published trial report, AE 1–2 were recorded, an objective response (OR) was achieved in 25% of cases, and it was also mentioned that personalized neoantigen vaccines have the ability to induce a T-cell response [61].

Koichi Mitsuya and colleagues used polarized α-type 1 DCs in a clinical trial as a vaccine against multifocal glioblastoma. DCs were activated with a cytokine cocktail and a complex of synthetic peptides. Combining the vaccine with standard therapy showed an anti-tumor effect, with an mOS of 19.0 months. The study also found significant IL-12 secretion by α-type DCs, which correlated with increased levels of IFN-γ, potentially inducing the IFN-γ-mediated stimulation of effector T-cells. The long-term follow-up of patients (up to 6 years) revealed better outcomes for those receiving DC therapy, compared to the control group [114].

#### 5.2.2. Therapy Based on Natural DCs

Clinical trial NCT02574377 evaluated immunological effects in patients with stage 3 resected melanoma, who had undergone autologous myeloid-derived cDC2 or pDC therapy. Cells were isolated by apheresis and loaded with melanoma-associated peptides. No third-grade or higher AE were observed, and antigen-specific CD8^+^ T-cells were present in 80% of patients, and CD4^+^ T-cells in 64%, following the first vaccine injection. Median disease-free survival (mDFS) was 19.4 months, and 6 out of 15 patients showed no recurrence at the end of the study [110]. In 2023, a phase 1/2 clinical trial of a neoadjuvant DC vaccine for ovarian cancer treatment was launched, with the selection of natural cDC1 as the most effective DC population for antigen cross-presentation as a therapy basis (NCT05773859)

#### 5.2.3. Therapy Based on DC-Derived Vesicles (Dex)

Dex vaccines have been tested in a series of clinical trials. In phase 1 of the Dex clinical trial, those obtained from autologous DCs were administered to patients with metastatic melanoma. Despite the promising method, only 2 out of 15 participants developed a PR, while SD was observed in two patients. However, no AE were identified, and 8 out of 13 individuals showed increased effector function of NK cells [115]. In another phase 1 clinical trial, autologous Dex therapy loaded with MAGE tumor antigens demonstrated MAGE-specific T-cell responses in 33% of patients with non-small cell lung cancer, as well as an increase in NK lytic activity in 16% of patients [116]. After the phase 2 study using Dex-stimulated IFN-γ, Dex was shown to enhance anti-tumor immunity in the NK cells of patients with advanced non-small cell lung cancer (NCT01159288). The results indicate that the Dex vaccine promotes the increased effector function of NKp30-NK cells, which is closely related to high levels of MHC II expression and IFN-γ content. No T-cell response was detected in patients. One patient developed third-grade hepatotoxicity, while, in other cases, AE did not exceed the second grade [117].

**Table 2:** Results from clinical trials using dendritic-cell-based therapeutic vaccines.

Therapy	Type of Cancer	Participants	Efficiency, %	References
Neoantigen-primed DC vaccine + cyclophosphamide	Non-small cell lung cancer	12	25 OR 75 DCR 100 AE (1–2)	NCT02956551
Adenoviral transduced autologous human epidermal growth factor receptor (AdHER)/neu DC vaccine	Metastatic solid tumors characterized by HER2/Neu expression	33	3 CR 3 PR 15 SD 100 AE	[116]
Autologous DCs transduced with AdGMCA9 (DC-AdGMCAIX)	Metastatic renal cell carcinoma	11	45 AE (1–2) 9 SD	NCT01826877
Autologous DCs pulsed with tumor lysate antigen (DCVax^®^-L)	Newly diagnosed glioblastoma (NDG); recurrent glioblastoma (RG)	232—NDG 64—RG	1,5 AE 19.3 mOS (months), NDG 13.2 mOS (months), RG	NCT00045968
IKKβ-matured RNA-transfected DC vaccine + immune checkpoint blockade (ICB)	Metastatic uveal melanoma	12	No published results	NCT04335890
Autologous DCs + docetaxel + prednisone	Metastatic castration-resistant prostate cancer	1182	23.9 mOS (months) 24.3 mOS (months, placebo group)	NCT02111577
Alpha-type-1 polarized DC-based vaccination	Newly diagnosed high-grade glioma	16	19 mOS (months)	[114]
DCs transduced with MART-1, tyrosinase, and MAGE-A6+ IFN-α	Melanoma	35	5.7 PR 23 SD 40 PD 51.4 AE 36 mOS (months)	NCT01622933
Therapy based on natural autologous cDC2 and pDCs	Melanoma	15	19.4 mDFS (months) 100 AE (1–2)	NCT02574377
Exosomes derived from autologous moDCs	Metastatic melanoma	15	13 PR 13 SD	[58]
IFN-γ-exosomes derived from DC (IFN-γ- Dex)	Non-small cell lung cancer	22	15 mOS (months) 19 AE (1–3) 32 SD (for 4 months)	NCT01159288

OR—objective response, DCR—disease control rate, AE—adverse events, CR—complete response, PR—partial response, SD—stable disease, mOS—median overall survival, PD—progressive disease, mDFS—median disease-free survival.

## 6. Conclusions

Despite the discouraging results of DC-based immunotherapy approaches, addressing the challenge of enhancing the clinical response remains relevant. Vaccines may be a proper treatment choice for individuals with tumors non-responsive to CAR-T therapy, immune checkpoint inhibitors, or monoclonal antibodies and may also be suitable for maintaining remission. The analysis of different populations of DCs in the human body provides a clear understanding of their contribution to the mechanisms of immune function—in particular, anti-tumor processes. Among preclinical studies, there is a trend towards using generated cDCs and pDCs as immunotherapy, demonstrating promising results. However, obtaining sufficient quantities of DCs from precursors in vitro to achieve a clinical response in patients remains an open question.

In conclusion, it should be noted that numerous clinical trials aiming to test combined treatment options are currently either in the patient recruitment stage or have not been completed yet. Although DC-based vaccines face a number of difficulties, recent advances in the development of new DC vaccination schemes as adjuvant therapy will make a significant contribution to therapy, especially for solid tumors. It is likely that the new generation of DC vaccines based on mRNA loading and combined with various immune adjuvants will lead to a significant T-cell response and a reduction in the influence of the TME. An alternative and promising therapy option is the direct generation of cDC1 from the patient’s blood as the most effective APC for an anti-tumor response. The most important steps in improving DC vaccines are optimizing the methods of differentiating DCs from precursor cells, selecting the most effective method of loading, and combining DC vaccines with other forms of therapy, which may contribute to increasing their effectiveness and achieving the best clinical outcome for cancer patients.

## Data Availability

Authors can confirm that all relevant data used to support the findings of this study are including within the article.
